# microRNAs profiling of small extracellular vesicles from midbrain tissue of Parkinson’s disease

**DOI:** 10.3389/fnmol.2023.1090556

**Published:** 2023-02-03

**Authors:** Zhengzhe Li, Dongdong Chen, Renjie Pan, Yanbiao Zhong, Tianyu Zhong, Zhigang Jiao

**Affiliations:** ^1^The First School of Clinical Medicine, Gannan Medical University, Ganzhou, China; ^2^Laboratory Medicine, First Affiliated Hospital of Gannan Medical University, Ganzhou, China; ^3^Department of Rehabilitation Medicine, First Affiliated Hospital of Gannan Medical University, Ganzhou, China; ^4^Precision Medicine Center, First Affiliated Hospital of Gannan Medical University, Ganzhou, China

**Keywords:** small extracellular vesicles, microRNAs, Parkinson’ disease, midbrain tissue, biomarkers

## Abstract

Small extracellular vesicles (sEVs) are generated by all types of cells during physiological or pathological conditions. There is growing interest in tissue-derived small extracellular vesicles (tdsEVs) because they can be isolated from a single tissue source. Knowing the representation profile of microRNA (miRNA) in midbrain tissue–derived sEVs (bdsEVs) and their roles is imperative for understanding the pathological mechanism and improving the diagnosis and treatment of Parkinson’s disease (PD). bdsEVs from a rat model of PD and a sham group were separated and purified using ultracentrifugation, size-exclusion chromatography (SEC), and ultrafiltration. Then, miRNA profiling of bdsEVs in both groups was performed using next-generation sequencing (NGS). The expression levels of 180 miRNAs exhibited significant differences between the two groups, including 114 upregulated and 66 downregulated genes in bdsEVs of PD rats compared with the sham group (*p* < 0.05). Targets of the differentially expressed miRNAs were predicted by miRanda and RNAhybrid, and their involvement in the signaling pathways and cellular function has been analyzed through the Kyoto Encyclopedia of Genes and Genomes (KEGG) pathway and Gene Ontology (GO). Furthermore, we explored the expression levels of miR-103-3p, miR-107-3p, miR-219a-2-3p, and miR-379-5p in bdsEVs, sEVs derived from plasma, and plasma of both groups of rats. Interestingly, the expression levels of miR-103-3p, miR-107-3p, miR-219a-2-3p, and miR-379-5p were elevated in bdsEVs and sEVs from plasma; in contrast, their expression levels were decreased in plasma of the rat model of PD. In summary, miRNAs may play a significant role in the onset and development of PD, and miRNAs need to be selected carefully as a research subject for exploring the pathological mechanism and the potential therapeutic targets and diagnostic markers of PD.

## 1. Introduction

Parkinson’s disease (PD), the second most common neurodegenerative disorder (NDD) just after Alzheimer disease (AD), is more usual in the seniors, with a mean onset age of about 60 years (Tysnes and Storstein, 2017). The major clinical symptoms of PD encompass impaired movement function, including resting tremor, rigidity, and loss of postural reflexes. The pathological hallmark of PD is the formation of Lewy bodies containing α-synuclein (α-syn) aggregates in tyrosine hydroxylase-positive (TH+) neurons (Yousefi et al., 2022). Although studies in animal models and patients with PD have indicated that the transmission of neurotoxic molecules between neural cells plays a vital role in the progression of PD, the underlying molecular mechanisms are still unclear (Hirsch et al., 2013). Therefore, it is a priority to comprehend these processes and mechanisms in PD, so as to assist in developing better prevention and treatment interventions.

microRNAs (miRNAs) are noncoding single-stranded small RNAs composed of 18–25 nucleotides, and play a main role in modulating gene expression (Krol et al., 2010). miRNAs are important in cell differentiation, biological function, and disease development; they are also essential in the function of dendrites, synaptic plasticity, and normal cognitive capability in the brain (Bartel, 2018). For example, previous studies have shown that the brain expression levels of many miRNAs are significantly altered in different NDDs, including AD, amyotrophic lateral sclerosis (ALS), and multiple sclerosis (MS), which can be used for differential diagnosis of PD (Sheinerman et al., 2017; Konovalova et al., 2019; Wang and Zhang, 2020). In addition, researchers have also found that miRNAs can be directly regulated during the development of PD. For instance, it has been found that miR-34b and miR-34c decrease the expression of α-syn in SH-SY5Y (Kabaria et al., 2015), and miR-494 exacerbates MPTP-induced neurodegeneration by downregulating DJ-1 (Xiong et al., 2014). Our previous study showed that the expression of miR-543-3p was obviously increased in astrocytes treated with MPP^+^, which could straightly regulate the function of glutamate transporter-1 (GLT-1), suggesting that an miR-543-3p inhibitor may be a new therapeutic drug for PD (Wu et al., 2019). Therefore, it is necessary to identify miRNAs in a variety of sources such as tissues and biological fluids, including saliva, plasma, serum, and urine, which could be used as potential biomarkers for PD.

Small extracellular vesicles (sEVs) are heterogeneous membranous vesicles (30–200 nm) that are secreted into the extracellular environment by all types of cells through different biogenetic and excretive mechanisms (Fabbiano et al., 2020). For example, α-syn–containing sEVs released by microglia can induce α-syn aggregation in recipient neurons to cause PD (Guo et al., 2020). According to de Rus Jacquet et al. (2021), LRRK2 G2019S sEVs secreted by astrocytes in PD are abnormally enriched in neurites and reduce neurotrophic support to dopaminergic neurons. In addition, sEVs miR-137 induces oxidative stress injury *in vitro* and PD-like motor function injury *in vivo* by downregulating OXR1 (Jiang et al., 2019). Another study showed that sEVs miR-106b targeted CDKN2B in neurons, which reduced neuronal apoptosis and increased neuronal autophagy in PD (Bai et al., 2021). Based on the sources from which they are obtained, sEVs can be typically classified into the following three types: cell culture–derived sEVs, body fluid–derived sEVs, and tissue-derived sEVs (tdsEVs; Li et al., 2021). sEVs contain the contents originating from their parent cells, thus partially representing the physiological and pathological status of the parent cells (Cocucci and Meldolesi, 2015; Huang et al., 2021). Therefore, sEVs derived from cultured cells or body fluid and their contents have been extensively investigated because they are both more easily accessible than midbrain tissue–derived sEVs (bdsEVs) in PD (Guo et al., 2020; He et al., 2021). These studies have extended our knowledge about PD; however, they have certain limitations. For instance, sEVs derived from cultured cells do not fully reflect the state of brain tissue because they lack stimulation or cytokines from other cells. Moreover, sEVs derived from body fluids are a mixture of many sources and are not specific for brain tissue. Hence, these limitations have resulted in inconsistency and irreproducibility of the conclusions.

As tdsEVs are most likely to act locally, they are considered to more accurately reflect the physiological and pathological state of the corresponding organs compared with sEVs derived from cell culture and body fluid (Crescitelli et al., 2020). miRNAs, one of the components of sEVs, are able to directly reflect the conditions of the central nervous system (CNS; Kalluri and LeBleu, 2020). Therefore, we focused on the miRNAs in bdsEVs from rat models of PD to explore the mechanism of action of miRNAs in PD and their capacity as potential diagnostic biomarkers. In our present study, we established an experimental rat model of PD by rotenone, used next-generation sequencing (NGS) to characterize the expression profile of miRNA, and determined the difference in miRNAs profile between bdsEVs from PD and sham rats.

## 2. Materials and methods

### 2.1. Animals and treatment

Adult male Sprague–Dawley (SD) rats (weight, 250 g) were purchased from Hunan SLAC Laboratory Animal Co., Ltd. (Changsha, China). The Guide for the Care and Use of Laboratory Animals was followed, and the study was approved by the Institutional Animal Care and Use Committee of Gannan Medical University. The rats were housed in cages under 12 h light/dark cycles with free access to food and water.

### 2.2. Rotenone-induced lesions

The rats were anesthetized using an intraperitoneal (IP) injection of ketamine (100 mg/kg) and xylazine (10 mg/kg) and fixed on a stereotaxic (ST) instrument (RWD Life Science, Shenzhen, China) in a flat position. They received bilateral injections of rotenone/vehicle in 2 μL (6 μg/μL) volumes (injection velocity of 0.20 μL/min) into the right side of the striatum at the following coordinates: anterior +0.24 mm, lateral ±3.6 mm, and ventral −4.5 mm. We removed the needle after 5 min to ensure the drug diffusion. The rats were put on a heating plate before waking up and then returned to their cages.

### 2.3. Balance beam test

Balance beam test was used to evaluate the motor balance of the rats as described previously (Feng et al., 2014). The apparatus was equipped with a 100-cm-long and 7-cm-wide beam placed horizontally at 100-cm-height above the floor. The rats were permitted to cross the beam to the dark home cages from one end to the other end. The times of paw slippage and the time of walk across the beam were recorded.

### 2.4. Rotarod test

An accelerating rotarod test (YLS-31A, Jinan Yiyan Technology Development Co., Ltd.) was used to evaluate motor coordination of the rats as reported previously (Deacon, 2013). The rats were tested three times in one experiment, 5 min apart. The rotarod bar was set to accelerate from 10 to 40 rpm in a 5 min period. The time that the rats were able to stay on the rotating rod was recorded.

### 2.5. Pole test

Pole test was conducted as reported previously (Ju et al., 2010). Briefly, the rats were put on the apex of the pole (diameter, 2.5 cm; height, 100 cm) with their head straight down. The time of climbing down to the underside of the pole until the feet reached the floor was recorded for locomotion activity (T-LA). Each rat was assessed three times.

### 2.6. Traction test

Limb movement coordination was assessed by the traction test, as reported previously (Jiao et al., 2020). A stainless-steel bar (diameter, 1.5 mm; length, 30 cm) was fixed 70 cm over the base. The rats were hung by their forefeet and left on the bar, and latency until they fell was recorded. Each rat was assessed three times, with a 5 min resting interval between the trials.

### 2.7. Grip strength test

The grip strength of forefeet was measured using a rat Grip Strength Meter with a sensor range of 0–5,000 grams (g) (Columbus Instruments, Columbus, OH). The maximum of three pulls of the Grip Strength Meter conducted between 9 and 11 a.m. was recorded; the test was performed daily over three consecutive days, and the resulting three daily maximum values were averaged (Jacobs et al., 2017).

### 2.8. Open field test

A square arena (100 cm × 100 cm) with 40-cm-high walls and white floor was used for the open field test. The arena had a central zone (25 cm × 25 cm) and a peripheral zone. The rats were put in the center of the arena and observed for 10 min. The behavioral total distance were recorded with a video camera, and Any-Maze behavioral tracking software was used for analysis. The arena was cleaned by 75% ethanol after each trial.

### 2.9. Immunofluorescent assay

Immunofluorescent assay was performed as described previously (Zhang et al., 2017). Sections were cut into 10-μm-thick slices, treated with phosphate buffered saline (PBS), and then incubated in 5% bovine serum albumin (BSA) for 60 min. The sections were incubated with primary antibodies at 4°C overnight, rinsed with PBS, and incubated with Alexa Fluor 488–conjugated goat anti-rabbit IgG for 1 h at room temperature. DAPI was used to stain cellular nuclei. Immunostaining was imaged using a ZEISS lsm 880 laser-scanning confocal microscope (ZEISS, Germany). Analysis of fluorescent intensities and particle features was performed by ZEISS software (ZEISS, Germany).

### 2.10. Separation of sEVs from midbrain tissue

The rats were anesthetized with an IP injection of ketamine and xylazine. Then, thoracotomy was performed to extract brain tissue by injecting 50 ml saline from the left ventricle. The midbrain tissues were stored at −80°C. For bdsEVs separation, we used a Tumor Dissociation Kit, human (Miltenyi Biotec, 130-095-929) following the manufacturer’s protocol. The isolation was reported as described previously (Vella et al., 2017; Chen et al., 2021; Crescitelli et al., 2021). The midbrain tissues were cut into small pieces and weighed (~200 mg) with a cryostat, then placed in 2.2 mL DMEM/F-12 medium (Gibco, C11330500BT) containing 100 μL Enzyme H, 50 μL Enzyme R, and 12.5 μL Enzyme A, and incubated at 37°C for 15 min. After digestion, the samples were placed on ice immediately. The samples were filtered through a 70-μm cell strainer (Biosharp, BS-70-XBS) twice to remove larger tissue debris. The filtered liquids were treated by differential centrifugation (300 *g*, 10 min; 2,000 *g*, 10 min; 10,000 *g*, 20 min) and 0.22-μm filtration (Millipore, SLGPR33RB) to remove cellular fragments, bigger vesicles, and any cluttered particles. The supernatant was ultracentrifuged at 150,000 *g* for 120 min, and the sediment was resuspended with 1 mL PBS. The supernatant was loaded into a Sepharose-based CL-2B column (ECHO BIOTECH, Echo9103A-10 mL) prewashed with 20 mL sterile PBS. bdsEVs and other fractions were eluted with PBS after all of the samples had entered into the column and no fluid flowed out from the column bottom. Each 500 μL of effluent represented one fraction. Four to seven fractions were gathered and added into a 100-kDa Amicon Ultra-15 ultrafiltration tube (Millipore, UFC910024). The enriched bdsEVs were gathered for experiments after centrifuge at 4,000 *g* for 20 min. Temperature for all centrifugations was 4°C.

### 2.11. Transmission electron microscopy

We dropped 10 μL of bdsEVs sample onto the copper mesh, and cleaned it with sterile distilled water after incubation for 10 min at room temperature. Then, 10 μL of 2% uranyl acetate was dropped for 1 min for negative staining, followed by drying for 2 min under an electric bulb. Grids were imaged with a transmission electron microscope (H-7650, Hitachi Ltd., Tokyo, Japan).

### 2.12. Nanoparticle tracking analysis

Full bdsEVs were diluted with PBS to 1 × 10^7^–1 × 10^9^ mL and quantified with the ZetaView PMX 110 (Particle Metrix, Meerbusch, Germany). A 60 s video was shot at 30 frames/s under 405 nm emission light, and the particle trajectory was analyzed with NTA software (ZetaView 8.02.28) to obtain the size and concentration of bdsEVs.

### 2.13. Western blotting

We used BCA protein quantitative kit to measure the protein concentrations of bdsEVs and tissues. The protein content of each sample was adjusted to 10–30 μg and added to 5 × SDS buffer, and the proteins were denatured in 95°C water bath for 5 min before electrophoresis. The proteins were transferred to PVDF membranes at low temperature using a constant current of 200 mA. The membranes were blocked with 3% BSA blocking solution at room temperature for 1 h, and then incubated at 4°C overnight with antibodies against TH (66334-1-Ig, Proteintech, Rosemont, IL), HSP70 (60318-I-Ig, Proteintech, Rosemont, IL), CD9 (60232-I-Ig, Proteintech, Rosemont, IL), TSG101 (sc-13,611, Santa Cruz, CA, United States), and calnexin (10427-2-AP, Promega, Madison, WI). They were incubated with secondary antibodies at room temperature for 1 h before development, fixing, and exposure.

### 2.14. RNA isolation and library preparation

Total RNA was extracted from bdsEVs using miRNeasy^®^ Mini kit (Qiagen, cat. No. 217004) in accordance with the kit’s instruction. For a long-strand RNA library, the input amount of total RNA was 250 pg–10 ng, and the database was built through SMARTer Starveto Total RNA-Seq Kit (Takara Bio USA, Inc.). For the small RNA library, the input amount of each sample was 1–500 ng, and the library construction kit was QIAseq miRNA Library Kit (Qiagen, Frederick, MD). Library quality was assessed by Agilent 2,100 Bioanalyzer (Agilent Technology, United States) and qPCR. The index labeled samples were used to generate clusters in the acBot Cluster Generation system through TruSeq PE Cluster Kitv3-cBot-HS (Illumina, San Diego, CA, United States). Paired-ended sequencing was executed on an Illumina NovaSeq6000 platform. Sequencing and analysis of miRNAs were executed by ECHO Biotech Co., Ltd. (Beijing, China).

### 2.15. Bioinformatic analysis

The original image file (BCL) obtained by sequencing was converted into raw data (raw reads) in FASTQ format after base-calling. In order to gain clean reads, low-quality reads and <15 or >35 nt reads were filtered. For acquiring unannotated RNAs that contained clean reads, Bowtie alignment (Langmead et al., 2009) was done according to Silva, GtRNAdb, Rfam, Repbase, and NCBI. miRbase (v22) (Kozomara et al., 2019) and Mirdeep2 (Friedländer et al., 2012) were used to identify known and new miRNAs included in the unannotated RNAs. EdgeR (Anders and Huber, 2010) was used to calculate the *p*-value in biologically repeated experiments. Significantly differential expression of miRNAs was verified according to the criterion *p* < 0.05, |log2(FC)| ≥ 0.58, and TPM > 10. The Kyoto Encyclopedia of Genes and Genomes (KEGG) and Gene Ontology (GO) enrichment analyses were performed for differentially expressed miRNA target genes, and *p*-value was calculated using hypergeometric distribution.

### 2.16. RT-qPCR

Single-stranded cDNA was prepared in a 20 μL reaction solution using 1 μg of total RNA as template with PrimeScript™ RT reagent Kit (Perfect Real Time; TAKARA, RR037A) in line with the manufacturer’s protocol. We diluted the RT reaction mixture by a factor of 5 in nuclease-free water and stored it at −20°C.

A total of 20 μL of the reaction mixture was made up of 2 μL of cDNA, 10 μL of 2 × Premix Ex Taq™ (Probe qPCR; TAKARA, RR390A), 4 μL of nuclease-free water, 2 μL of microRNA-specific primer, and 2 μL of universal primer to synthesize appropriate DNA using Applied Biosystems^®^ QuantStudio^®^ 5 Real-Time PCR Instrument (Thermo Fisher Scientific Inc., United States). The assay was performed in the Axygen^®^ 0.2 mL Polypropylene PCR 8 Tubes Strip (Axygen, United States) at an initial 95°C for 30 s, followed by 50 cycles each at 95°C for 5 s and 58°C for 30 s. Each sample was run in triplicate. The primer and probe sequences used in the study are presented in the following table:

**Table:** 

Name	Primer/probe sequence(5′–3′)
qPCR-TYR	GTGCAGGGTCCGAGGT
U6-RT	AACGCTTCACGAATTTGCGT
U6-S	CTCGCTTCGGCAGCACA
U6-A	AACGCTTCACGAATTTGCGT
U6 probe	AGAAGATTAGCATGGCCCCTGCGCA
rno-miR-103-3p-RT	GTCGTATCCAGTGCAGGGTCCGAGGTATTCGCACTGGATACGACTCATAG
rno-miR-103-3p-F	ACGAGCAGCATTGTACAGG
rno-miR-103-3p-PROBE	TTCGCACTGGATACGACTCATAGC
rno-miR-107-3p-RT	GTCGTATCCAGTGCAGGGTCCGAGGTATTCGCACTGGATACGACTGATAG
rno-miR-107-3p-F	ACGTCAGCAGCATTGTACAG
rno-miR-107-3p-PROBE	TTCGCACTGGATACGACTGATAGCC
rno-miR-219a-2-3p-RT	GTCGTATCCAGTGCAGGGTCCGAGGTATTCGCACTGGATACGACACAGAT
rno-miR-219a-2-3p-F	ACGTAAGAATTGTGGCTGGA
rno-miR-219a-2-3p-PROBE	TTCGCACTGGATACGACACAGATG
rno-miR-379-5p-RT	GTCGTATCCAGTGCAGGGTCCGAGGTATTCGCACTGGATACGACCCTACG
rno-miR-379-5p-F	ACGACTGTGGTAGACTATGGAA
rno-miR-379-5p-PROBE	TTCGCACTGGATACGACCCTACG

We used the standard 2^–ΔΔCt^ method to quantify the expression of target microRNAs, with U6 as the reference microRNA.

### 2.17. Statistical analysis

Data analysis and presentation were based on R software (version 3.6.1) and GraphPad Prism 8 software. The significance of differences between the groups was analyzed by two-tailed unpaired *t*-test at *p* < 0.05.

## 3. Results

### 3.1. Rotenone decreases motor activity and induces degeneration of dopaminergic neurons in rats

**Figure 1:**
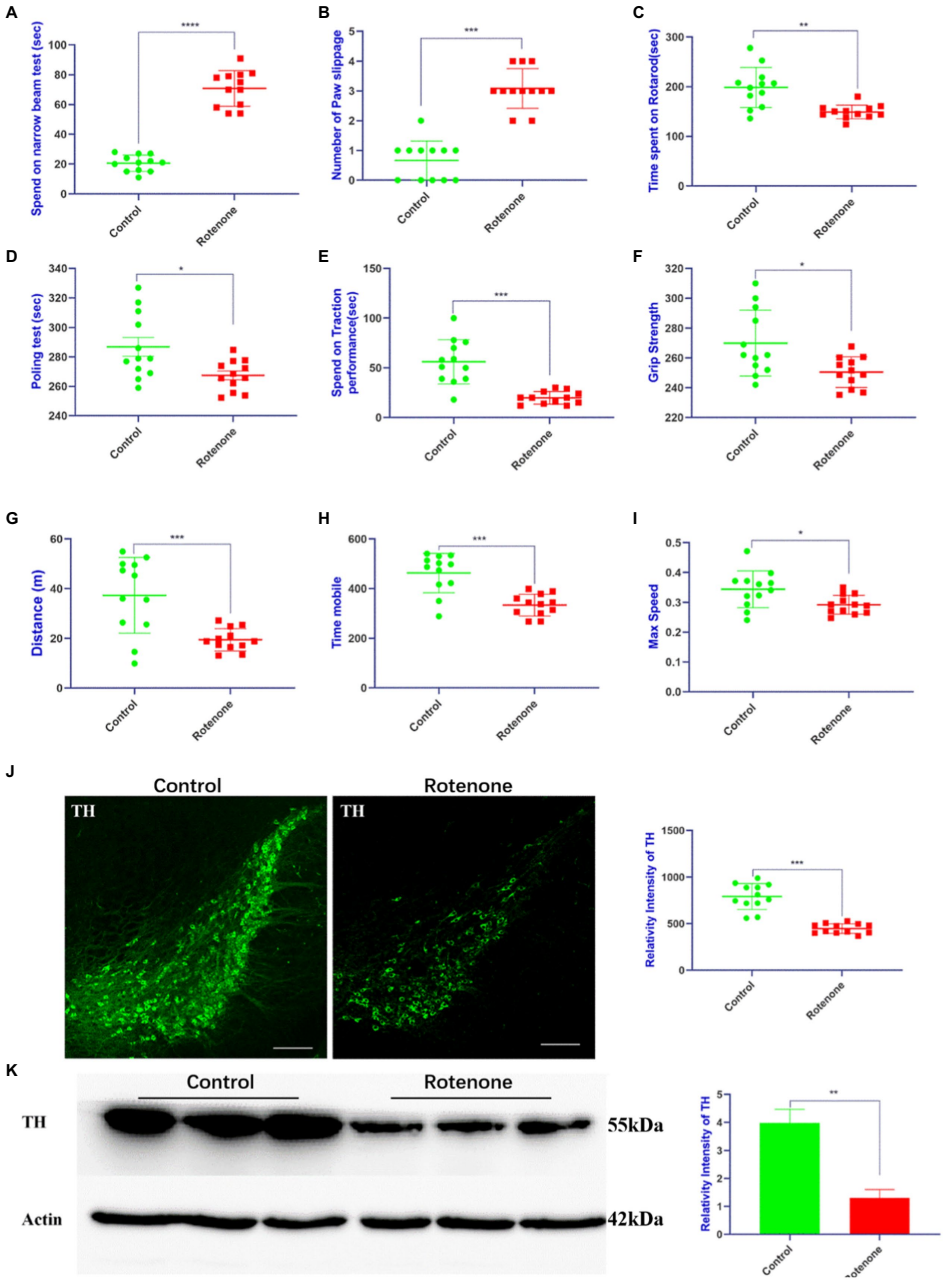
Rotenone induces motor deficits and DA neuron degeneration in rats. **(A,B)** Narrow beam walk test, **(C)** rotarod test, **(D)** pole test, **(E)** traction test, **(F)** grip strength test, and **(G–I)** open field test was performed at 28 days after rotenone/vehicle treatment through stereotaxic (ST) striatum injections (*n* = 12). **(J)** Left panel is visualized at ×5 magnification of TH-positive cells in the SNpc of rats stained using a TH antibody; right panel is the quantitative analysis of TH-positive cells in the SNpc. **(K)** Left panel is the Western blotting of midbrains separated from rats performed by a TH antibody and right panel is quantitative analysis of TH intensity. For all quantitative/statistical analysis, *p < 0.05; **p < 0.01; ***p < 0.001 and ****p < 0.0001. All data are presented as means ± SEMs. Scale bars are 250 μm in **(J)**. DA, dopaminergic; TH, tyrosine hydroxylase; SNpc, substantia nigra pars compacta.

To establish a rat model of PD, rotenone/vehicle was injected into the striatum of the rats with ST. At 28 days after injection, behavioral tests including motor activity and coordination were performed to assess the movement function of the rats. The behavioral assessments revealed that the rotenone-injected rats took more time (Figure 1A) to traverse the beam and exhibited the maximum number of paw slipping (Figure 1B), less time spent on the rotarod, pole test, and traction performance compared with vehicle-treated rats (Figures 1C–E). Additionally, the rotenone-injected rats exhibited lower grip strength (Figure 1F) and less distance traveled, mobile time, and maximum speed (Figures 1G–I) in the open field. Compared with the vehicle-treated rats, the resisting arrest behavior was weakened, indicating that the motor skills of the rats had been reduced by rotenone treatment. Overall, the impaired motor function in the rats was revealed by these behavioral results after rotenone exposure. In addition, we found that tyrosine hydroxylase (TH)–positive cells and TH protein expression were obviously decreased in the substantia nigra pars compacta (SNpc) of the rotenone-treated rats compared with the vehicle-treated rats (Figures 1J, K). Taken together, these results suggest that rotenone induces PD-like behavioral deficits and degeneration of dopaminergic (DA) neurons in SNpc of rats.

### 3.2. Characterization of midbrain tissue-derived sEVs enriched fractions

**Figure 2:**
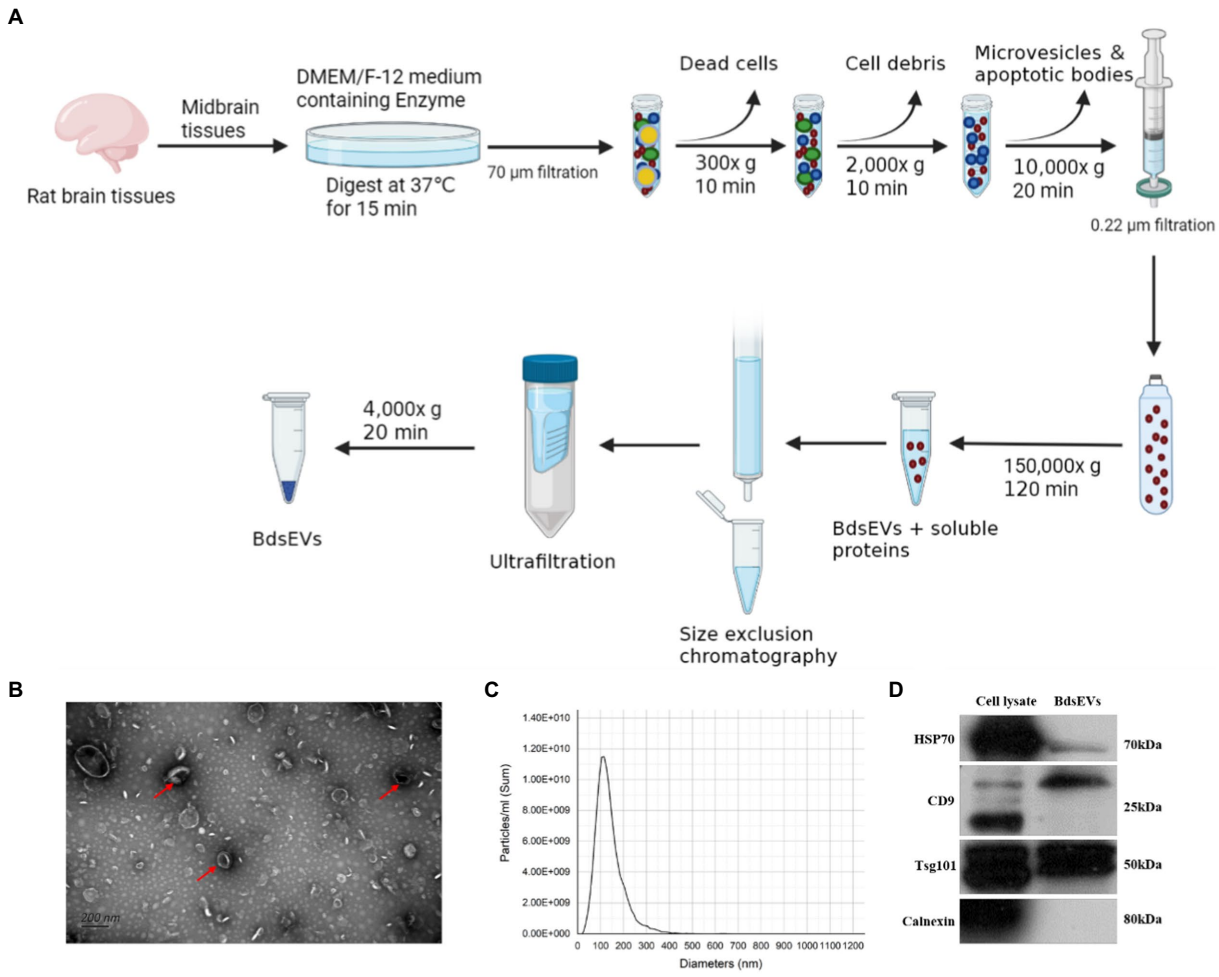
Isolated midbrain tissue–derived sEVs (bdsEVs) enriched fractions. **(A)** Isolation overview, **(B)** Transmission electron microscopy (TEM) images of bdsEVs, **(C)** Nanoparticle tracking analysis (NTA) of bdsEVs, and **(D)** sEVs positive markers HSP70, CD9, and TSG101; negative marker calnexin of bdsEVs enriched fractions isolated from the midbrain. sEVs, small extracellular vesicles; HSP70, heat-shock protein 70; CD9, cluster of differentiation 9; TSG101, tumor susceptibility gene 101.

To gain the midbrain tissue–derived sEVs (bdsEVs) enriched fractions from the midbrain tissue, we first digested the midbrain tissue from both groups of rats. Then, ultracentrifugation, size-exclusion chromatography (SEC), and ultrafiltration were performed gradually to isolate and purify bdsEVs (Figure 2A). Then, 200-μL bdsEVs fractions were acquired from 200-mg midbrain tissue, and the concentration of bdsEVs (1.3 × 10^13^ particles/mL) was calculated by NTA. In order to observe the form of bdsEVs, the negatively stained samples of bdsEVs were observed by TEM. The structure of the monolayer membrane was very clear under an electron microscope; the vesicles were saucer-or disc-shaped with one flat side recessed, giving them the appearance of a bowl (Figure 2B). The size and concentration of bdsEVs were further determined by NTA analysis. The main particle size peak of bdsEVs was at 113.3 nm, and 87.7% of the particles had a size between 50 and 200 nm (Figure 2C). Western blotting was used to detect the characteristic protein markers (HSP70, CD9, and TSG101) of bdsEVs (Figure 2D). Conversely, calnexin, absent in the bdsEVs enriched portion, was the negative biomarker of sEVs (Figure 2D). These results showed that the extracted bdsEVs were correctly characterized on the basis of the MISEV2018 Guide (Théry et al., 2018).

### 3.3. Sequencing data of bdsEVs from rats

**Figure 3:**
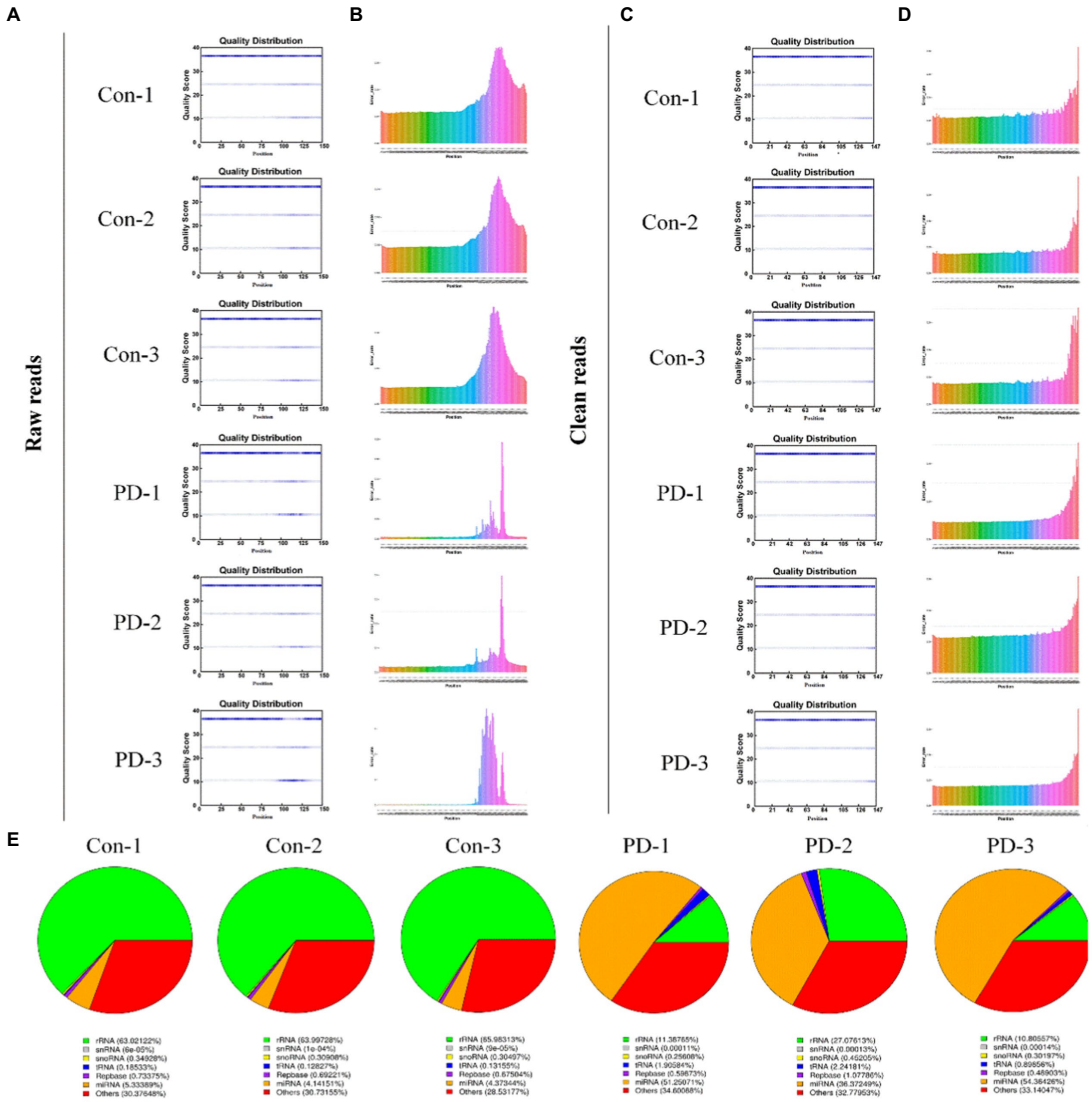
Sequencing data quality control. **(A,B)** Raw data quality control: **(A)** The base quality distribution box plot and **(B)** Base error rate in raw reads. **(C,D)** Clean data quality control: **(C)** The base quality distribution box plot and **(D)** Base error rate in raw reads. **(E)** RNA type of reads.

In this study, we constructed six cDNA libraries using total RNA from bdsEVs from rotenone-treated rats and DMSO-treated rats. The cDNA library raw reads of different samples were as follows: Con-1, 35,915,602; Con-2, 29,840,207; Con-3, 25,226,379; PD-1, 25,951,856; PD-2, 33,348,463; and PD-3, 28,719,407. GC content (%) percentages were as follows: 56.73 in Con-1; 57.45 in Con-2; 58.24 in Con-3; 55.1 in PD-1; 55.99 in PD-2; and 55.07 in PD-3 (Supplementary Table S1). We performed a quality control analysis of the raw data. The base quality of the raw reads was counted during each sequencing cycle. The blue line in Figure 3A represents the average baseline mass of the cycle in the baseline mass distribution box plot. As shown in Figure 3B, the basic error rate of the raw read was limited to 0.021–0.025. Clean data were obtained by filtering the raw data. The statistics of clean reads, GC%, Q20, Q30, and clean bases are shown in Supplementary Table S2. The quality control results of clean reads are shown in Figures 3C,D. In addition, by extending the BWT and Ferragina–Manzini (Fm) indexing algorithms and aligning the clean reads with the rat reference genome using HISAT2 software, it was determined that the total mapped reads or fragments of all samples were above 80% and mapped to the reference genome (Supplementary Table S3). The clean reads were analyzed using the distributions of the known RNA types (Figure 3E).

### 3.4. Profiling of differentially expressed miRNAs

**Figure 4:**
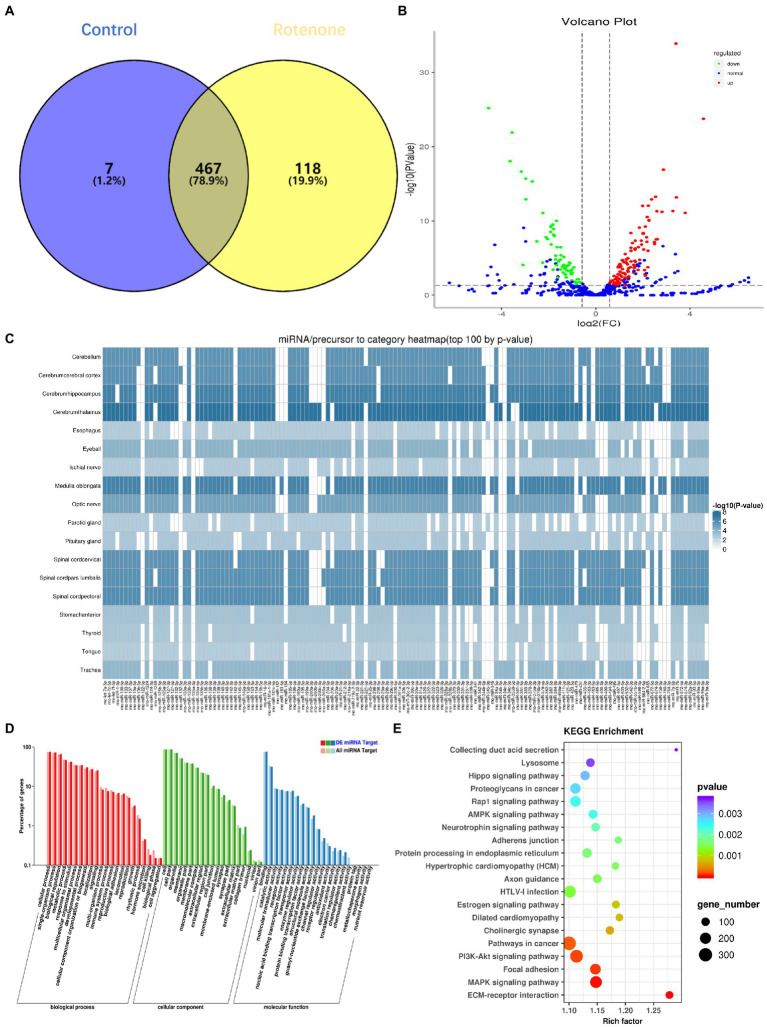
Differential microRNA (miRNA) expression between sham and Parkinson’s disease (PD) midbrain tissue–derived small extracellular vesicles (bdsEVs) and analysis of differentially expressed genes (DEGs) in PD bdsEVs. **(A)** The Venn diagram of 592 known mature miRNAs, **(B)** Volcano plot of differentially expressed miRNAs between sham and PD bdsEVs. The Y-axis of the graph represents significance (−log10 of the value of p) and the X-axis represents log2 of the fold change (expression in PD bdsEVs/expression in sham bdsEVs). Red, significantly upregulated miRNAs; green, significantly downregulated miRNAs; blue, no significant difference. Fold change >1.5 (log2 FC > 0.58 or <−0.58) and *p* < 0.05 (−log10 (*p*-value) > 1.3) were considered significant, **(C)** Heat map of differential top 100 miRNA precursor categories by *p*-value, **(D)** Gene Ontology (GO) enrichment analysis of CC, MF, and BP presenting the items of GO enrichment (*p* < 0.05 and FDR < 0.05), and **(E)** Kyoto Encyclopedia of Genes and Genomes (KEGG) pathway analysis of the KEGG pathways of DEGs in the sham and PD groups (*p* < 0.05 and FDR < 0.05). BP, biological processes; CC, cellular components; MF, molecular function; FDR, false-discovery rate.

There is emerging evidence that sEVs miRNAs function as important regulators of molecular functions in cells (Garcia-Martin et al., 2022). To identify the changes in miRNA expression, we used high-throughput sequencing (miRNA-seq) to profile the miRNAs in bdsEVs in three replicates; bdsEVs from the sham group as a comparative sample were profiled. A total of 474 and 585 known mature miRNAs were identified in PD-bdsEVs and Con-bdsEVs, respectively (Figure 4A). A total of 467 miRNAs were detected in both PD-bdsEVs and Con-bdsEVs (Figure 4A).

With a 1.5-fold change (|log2 (Fold Change)| > 0.58, *p* < 0.05, and TPM > 10), a total of 180 miRNAs in bdsEVs from the PD group were differentially expressed compared to the control group according to the cleansing of normalized sequencing data. Among them, 114 and 66 miRNAs were up-and downregulated, respectively (Figure 4B). An expression heat map (Supplementary Figure S1) demonstrates the normalized expression levels of the selected miRNAs following a hierarchical clustering method, where red and green represent the higher and lower levels of expression, respectively. To determine the correlation between these miRNAs and the brain, a heat map shown in Figure 4C was created to demonstrate the distribution of the top 100 miRNA categories in the brain by *p*-values. To define the potential functions and pathways regulated by these miRNAs, target predictions for the differentially expressed miRNAs were performed by miRanda and RNAhybrid, and the overlapped targets were annotated by GO (Ashburner et al., 2000) and KEGG (Kanehisa et al., 2004). GO analysis revealed that most of the miRNAs were enriched in biological processes (BPs) in response to stimulus, cellular component organization, or biogenesis and growth, while the synapse part was significant in cellular components (CCs). These miRNAs were also enriched in the molecular functions (MFs) of transporter activity, enzyme regulator activity, and receptor regulator activity (Figure 4D). The lysosome, AMPK signaling pathway, neurotrophin signaling pathway, axon guidance, and PI3K–Akt signaling pathway (Figure 4E), which reflect possible physiological processes and the potential regulatory mechanisms during PD, were revealed by KEGG pathway analysis within the top 20 enriched pathways. Taken together, these results show that these miRNAs may be involved in PD by modulating their targets.

### 3.5. Validation of differentially expressed candidate miRNAs

**Figure 5:**
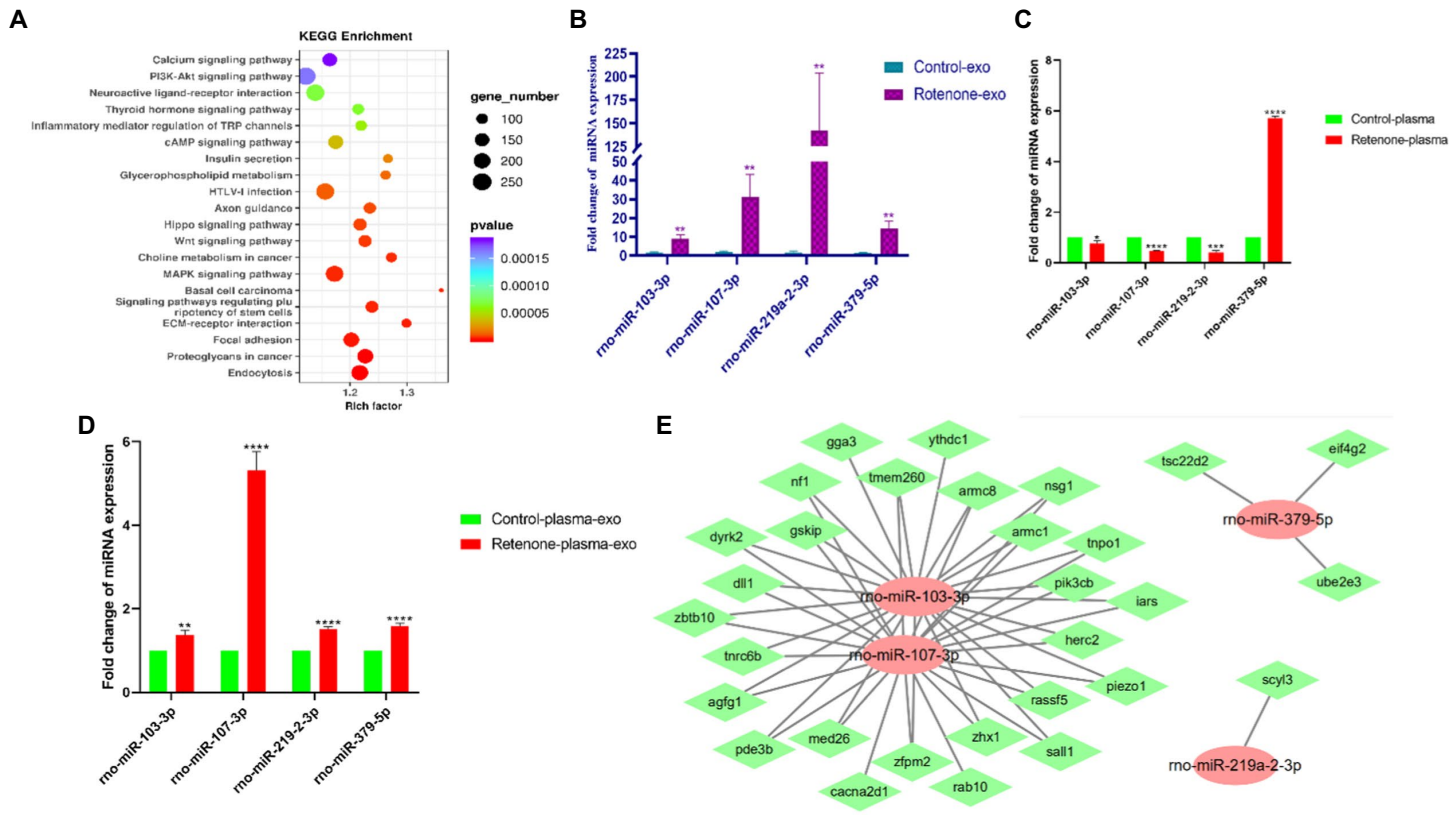
Validation of differentially expressed candidate miRNAs. **(A)** Kyoto Encyclopedia of Genes and Genomes (KEGG) pathway analysis of the top 25 KEGG pathways of obviously DEGs in the sham and PD groups (*p* < 0.05 and FDR < 0.05), **(B)** The expression levels of four midbrain tissue–derived sEVs (bdsEVs) miRNAs were analyzed in Parkinson’s disease (PD) (*n* = 3) and sham control (*n* = 3) using RT-qPCR, **(C)** The expression levels of the four miRNAs in plasma of Parkinson’s disease (PD) (*n* = 3) and sham control (*n* = 3), **(D)** The expression levels of the four miRNAs in sEVs derived from plasma of Parkinson’s disease (PD) (*n* = 3) and sham control (*n* = 3), and **(E)** The target gene predictions of the four bdsEVs miRNAs (rno-miR-103-3p, rno-miR-107-3p, rno-miR-219a-2-3p, and rno-miR-379-5p) by Targetscan (Total context++ score < −0.3), miRDB (Target Score > 75) and miRanda (Total score > 150, Total energy < −20). For all quantitative/statistical analysis, *p < 0.05; **p < 0.01; ***p < 0.001, and ****p < 0.0001. All data are presented as means ± SEMs. sEVs, small extracellular vesicles; RT-qPCR, reverse-transcription quantitative polymerase chain reaction; miR, microRNA.

To screen out miRNAs that correlate with PD and play a key role in the development of PD, we screened the top 25 miRNAs according to the concentrations and fold changes of miRNA expression (TPM > 10, *p* < 0.01, and |log2FC| > 2; Table 1). To analyze the potential functions and pathways regulated by these miRNAs, target predictions for the 25 miRNAs were performed by miRanda and RNAhybrid, and the overlapped targets were annotated by KEGG (Figure 5A).

**Table 1:** Top 25 differentially expressed microRNAs (miRNAs).

No.	miRNA_ID	(PD vs. Sham) log2 fold change	Value of *p*	FDR	Up/down
1	rno-miR-1839-5p	4.573149	1.73223E-24	3.77E-22	Up
2	rno-miR-31a-5p	3.421793	6.5997E-14	3.92E-12	Up
3	rno-miR-219a-2-3p	3.400552	1.28394E-34	8.38E-32	Up
4	rno-miR-328a-3p	3.163565	2.26002E-17	2.11E-15	Down
5	rno-miR-133b-3p	2.973991	1.9435E-16	1.59E-14	Down
6	rno-miR-328b-3p	2.967346	1.20762E-13	6.15E-12	Down
7	rno-miR-185-5p	2.869543	1.25035E-17	1.36E-15	Up
8	rno-miR-133a-3p	2.698304	4.78202E-16	3.47E-14	Down
9	rno-miR-1b	2.611429	2.89023E-08	4.49E-07	Up
10	rno-miR-15b-5p	2.591867	4.83307E-12	1.86E-10	Up
11	rno-miR-1-3p	2.565926	3.03393E-08	4.61E-07	Up
12	rno-miR-674-5p	2.535633	5.42603E-14	3.54E-12	Up
13	rno-miR-331-3p	2.500166	5.56761E-08	7.90E-07	Down
14	rno-miR-93-5p	2.368368	1.22466E-13	6.15E-12	Up
15	rno-miR-107-3p	2.258611	7.78938E-11	2.31E-09	Up
16	rno-miR-1,249	2.257513	4.22736E-05	3.06E-04	Down
17	rno-miR-103-3p	2.257107	8.62175E-11	2.45E-09	Up
18	rno-miR-127-3p	2.248478	8.46698E-12	2.76E-10	Down
19	rno-miR-379-5p	2.229127	8.91344E-13	4.14E-11	Up
20	rno-miR-298-5p	2.182619	2.92518E-11	9.10E-10	Up
21	rno-miR-140-3p	2.169973	6.37843E-08	8.68E-07	Up
22	rno-miR-497-5p	2.155196	1.55971E-08	2.55E-07	Up
23	rno-miR-322-5p	2.103942	5.61502E-10	1.31E-08	Up
24	rno-miR-664-2-5p	2.013669	4.24315E-09	7.92E-08	Up
25	rno-miR-664-1-5p	2.013011	5.38815E-09	9.77E-08	Up

In line with previous studies, miR-103-3p, miR-107-3p, miR-219a-2-3p, and miR-379-5p were further validated and analyzed by RT-qPCR. As expected, these four miRNAs in bdsEVs showed a similar expression trend to miRNA-sequencing data (Figure 5B). Subsequently, we detected the expression levels of these four miRNAs in plasma and sEVs derived from plasma of both groups of rats by RT-qPCR. Interestingly, the expression levels of miR-103-3p, miR-107-3p, and miR-219a-2-3p in the plasma of the rat model with PD were significantly lower compared with the sham group (Figure 5C). In addition, sEVs were separated from plasma, and the levels of these four miRNAs in sEVs were analyzed by RT-qPCR. As expected, the expression levels of these miRNAs in plasma sEVs were consistent with those of bdsEVs (Figure 5D). Finally, to determine the potential functions of target genes regulated by the four bdsEVs miRNAs (rno-miR-103-3p, rno-miR-107-3p, rno-miR-219a-2-3p, and rno-miR-379-5p), target gene predictions for these miRNAs were performed by Targetscan, miRDB, and miRanda (Figure 5E). The results suggested significant differences in the expression levels of miRNAs between plasma and sEVs from plasma, and the targets regulated by these miRNAs may contribute to the development of PD.

## 4. Discussion

miRNAs are stable in sEVs and are involved in many disease states by regulating disease-related genes or pathways (Kalluri and LeBleu, 2020). Although the mechanisms that regulate the selective secretion of miRNAs are not well understood, miRNAs have been shown to have potential value in the diagnosis, prognosis, and treatment of various diseases. In addition, uptake of miRNAs by receptor cells has been shown to have a biological function (Ying et al., 2017). miRNA patterns have been identified in sEVs extracted from body fluids of PD patients, and miRNA disorders have been found in sEVs extracted from the plasma or cerebrospinal fluid (CSF) of patients with idiopathic and hereditary PD (Jiang et al., 2021). For instance, Gui et al. (2015) found that miR-153, miR-409-3p, miR-10a-5p, and let-7 g-3p were significantly overexpressed; however, miR-1 and miR-19b-3p were significantly reduced in sEVs derived from CSF of patients with PD. Manna et al. (2021) reported that the levels of miR-22-3p and miR-223-5p in exosomes derived from serum of patients with PD were upregulated, while the level miR-21-3p was downregulated. Nevertheless, sEVs extracted from body fluids are a mixture of cells of different origin and have no specificity for brain tissue. Moreover, sEVs derived from cultured cells do not fully reflect the state of brain tissue due to the lack of stimulation or cytokines from other cells (Crescitelli et al., 2020). Compared with sEVs in body fluids, bdsEVs are first found in the interstitium of the brain tissue and most likely act locally. Thus, the composition of bdsEVs may reveal the physiological and pathological states of the brain. In addition, bdsEVs can be used to identify reliable cell-specific markers, which can then be used to capture specific populations of peripheral CNS-derived sEVs to aid in the diagnosis and monitoring of CNS disease. Moreover, bdsEVs samples contain minimal contaminants because they are from a single tissue (Jingushi et al., 2018). Analysis of bdsEVs at multiple disease sites and at different evolutionary stages may be helpful in discovering the process of disease development (Costa-Silva et al., 2015). However, miRNA profiling in bdsEVs from a rat model of PD or patients with PD has not yet been well defined. Hence, in the present study, we used rats treated with rotenone and analyzed the expression profile of miRNAs in bdsEVs derived from the rat model of PD.

In this study, we administered ST injection of rotenone into the striatum of the rats. After the completion of exposure, the behavioral tests and pathological array showed that rotenone induced PD-like behavioral deficits and degeneration of SNpc DA neurons, suggesting that these rats were appropriate research objects for our study. Therefore, we digested the tissue fragments; separated and purified bdsEVs by ultracentrifugation, SEC, and ultrafiltration; and then examined them by TEM, NTA, and western blotting. The results showed that separated sEVs derived from the midbrain tissue fulfilled the MISEV2018 Guide. Subsequently, NGS was used for miRNA profiling of bdsEVs derived from the rats, and we found distinct expression patterns between the PD and sham groups. As expected, 180 miRNAs showed significantly differential expression. Among them, the expression levels of 114 miRNAs were elevated, while the levels of 66 miRNAs were reduced. Through analysis, we found that the miRNAs in bdsEVs isolated from the brain tissue interstitial space were distributed centrally in the brain tissue, which can reflect the brain activity without interference with sEVs secreted by other tissues (Figure 4C). Then, 25 potential miRNAs were selected for further analysis according to the expression level combined with fold changes (TMP > 10, *p* < 0.01, and |log^2^FC| > 2) of miRNA in bdsEVs (Table 1). These differentially expressed miRNA–regulated target genes were enriched in the PI3K–Akt signaling pathway, axon guidance, neuroactive ligand–receptor interaction, MAPK signaling pathway, and Wnt signaling pathway as identified by KEGG pathway analysis (Figure 5A). As these pathways have been reported to be involved in the pathogenesis of PD by regulating neural function (Nataraj et al., 2017; Xue et al., 2019; Marchetti et al., 2020), our findings indicate that the miRNAs in bdsEVs may contribute to the etiology of PD.

Previous studies have shown that miR-103/107 forms a negative feedback loop with HMGA1 to regulate MPP^+^/MPTP-induced autophagy impairment and neural cell death (Li et al., 2020). Additionally, miR-103-3p inhibits neural stem cell proliferation and promotes apoptosis by targeting the Wnt/β-catenin signaling pathway through Ndel1 (Li et al., 2022). miR-107 is enriched in neurons compared with other cell types in the CNS, which is also predicted to target the neurodegeneration-related genes such as α-syn (Wang et al., 2014). Exosomes derived from neural stem cells are involved in neuroinflammation, apoptosis, and inhibition of neural regeneration *via* an miR-219a-2-3p–dependent mechanism (Ma et al., 2019), and overexpression of miR-219a-2-3p could promote apoptosis by suppressing MDM2 expression (Wang et al., 2020). miR-379-5p is related to fatty acid metabolism and metabolic pathways, and plays an important role in the pathophysiology of atherosclerosis and stroke (Niu et al., 2021). However, neuron–astrocyte coupling of fatty acid metabolism is essential for neuron survival (Ioannou et al., 2019; Qi et al., 2021), and fatty acid metabolism of neurons is also of great importance. As for the predicted target genes of these miRNAs, the target gene SCYL3 is involved in maintaining motor neuron viability (Kuliyev et al., 2018); eIF4G2 is involved in neuropathic pain (Zhang et al., 2021); and Rab10, NF-1, and Dll1 are indeed involved in PD (Imai et al., 2015; Kuo et al., 2021; Kluss et al., 2022). Many studies have demonstrated that these four miRNAs are involved in regulating brain function or brain damage in humans, mice, and rats. Therefore, rno-miR-103-3p, rno-miR-107-3p, rno-miR-219a-2-3p, and rno-miR-379-5p were selected for validation by quantitative PCR in bdsEVs, plasma, and sEVs from plasma in an independent rat model of PD. In line with the sequencing results, the expression levels of rno-miR-103-3p, rno-miR-107-3p, rno-miR-219a-2-3p, and rno-miR-379-5p in bdsEVs and sEVs from plasma were significantly elevated in the rat model of PD compared with the sham group. Interestingly, the expression levels of these miRNAs in plasma did not match with those in bdsEVs of PD rats. Although the expression levels of these miRNAs were increased in sEVs from plasma, their fold change expression was not as obvious as that of bdsEVs. The following reasons may be responsible for this inconformity: first, miRNAs in plasma are abundant, which could act on many organs and tissues; second, sEVs in plasma are derived from multiple organs compared with bdsEVs; lastly, the miRNA profiling of different organs shows a dramatic difference. These findings provide novel insights into sEVs miRNAs in PD, offering useful resources for the identification of PD biomarkers and possible therapeutic approaches for PD.

Although our findings partially represented the transcriptome of bdsEVs in PD, our study had several limitations. First, we only completed validation of the selected miRNAs in plasma and sEVs from plasma of PD rats in our study. miRNA expression profiling of sEVs derived from the plasma of PD patients was not performed, and we did not analyze the differences between the sEVs derived from the plasma of the control group and the PD patients. Considering this limitation, a question arises as to whether the results from RNA sequencing are applicable clinically. In further work, we will recruit patients with PD while expanding the sample size of animal models, and analyze the differences in miRNA expression between bdsEVs and sEVs from patients’ plasma, so as to promote the transformation of how sEVs miRNAs are applied in the clinic. Then, although we precisely dissected the midbrain tissue of the rats, it still contained a variety of nerve cells such as DA neurons, astrocytes, microglia, and so on. It is unclear which original cells released these differentially expressed miRNAs. We suggest that single-cell omics combined with sEVs transcriptomics may be a better solution to deal with this limitation. Most importantly, more work needs to be done to reveal sEVs miRNAs contribution to the pathogenesis of PD. To have a comprehensive understanding of PD sEVs biomarkers, we need to further explore the PD-associated sEVs miRNAs expression profiles of biological fluids, including plasma, serum, urine, and CSF.

## 5. Conclusion

In summary, we isolated and purified bdsEVs from the midbrain tissue of a rat model of PD and sham control, screened for miRNAs that are generally expressed in PD *via* NGS, and verified them by qRT-PCR assay. We found that the expression profiles of miRNAs in bdsEVs of PD model rats were significantly different from those of sham control rats. The expression of PD-related pathogenic genes or pathways is regulated by these abnormally expressed miRNAs directly or indirectly. A complicated regulatory network in PD pathogenesis is formed by the interactions between these miRNAs and their target genes. Hence, these miRNAs can be used as potential therapeutic targets for PD. These results lay the foundation for the pathogenesis and diagnosis of PD. Additionally, diagnosis, prevention, and treatment potential of miRNAs in bdsEVs should be further studied. Most importantly, our findings suggest that the expression profiles of miRNAs in sEVs and bdsEVs do not match with those in plasma, so caution is necessary when miRNAs in plasma alone are considered as biomarkers.

## Data availability statement

The datasets presented in this study can be found in online repositories. The names of the repository/repositories and accession number(s) can be found at: https://www.ncbi.nlm.nih.gov/, PRJNA891457.

## Ethics statement

The animal study was reviewed and approved by Institutional Animal Care and Use Committee of Gannan Medical University. Written informed consent was obtained from the owners for the participation of their animals in this study.

## Author contributions

ZJ and TZ conceived the study idea and designed the work. ZL, DC, and RP analyzed, interpreted the data, and provided partial samples. ZL and ZJ wrote the manuscript. YZ revised the manuscript critically. All authors contributed to the article and approved the submitted version.

## Funding

This work was supported by the Key Laboratory of Prevention and Treatment of Cardiovascular and Cerebrovascular Diseases, Ministry of Education (XN202014).

## Conflict of interest

The authors declare that the research was conducted in the absence of any commercial or financial relationships that could be construed as a potential conflict of interest.

## Publisher’s note

All claims expressed in this article are solely those of the authors and do not necessarily represent those of their affiliated organizations, or those of the publisher, the editors and the reviewers. Any product that may be evaluated in this article, or claim that may be made by its manufacturer, is not guaranteed or endorsed by the publisher.
