# Supplementary material for: microRNAs profiling of small extracellular vesicles from midbrain tissue of Parkinson’s disease
Source: Front Mol Neurosci. 2023 Feb 3;16:1090556. doi: 10.3389/fnmol.2023.1090556 (PMC9935574; doi:10.3389/fnmol.2023.1090556)
Supplement: Supplementary file 1 [file Data_Sheet_1.DOCX]

**Table S1** | Statistics of raw data quality



Total reads: statistics of total reads, each of the four adjacent lines contains the information read once, and the total number of reads per file is calculated; GC%: the percentage of G and C in all bases; Q20, Q30: percentage of total base numbers with Phred scores greater than 20 and 30(indicating basic call accuracy).

**Table S2** | Summary of filter data





Raw reads: the count of the original reads, each of the adjacent four lines contains information about one read, and the total number of reads per file is calculated; raw base: the number of all bases in raw data; clean reads: filtered to remove linker sequences, contaminated parts, and sequences containing more low-quality bases for a clean read; GC%: the percentage of G and C in all bases; Q20, Q30: percentage of total base numbers with Phred scores greater than 20 and 30(indicating basic call accuracy); clean bases: the number and length of sequences in clean reads calculate the number of bases; clean bases%: percentage of clean bases/raw base.

**Table S3** | Summary of reads mapped to the reference genome





Effective reads: the number of clean reads remaining after removal of rRNA reads will be used for subsequent genomic alignment; total mapped: The number of sequenced sequences that can be arranged on the genome; reads map to “+”: number of reads aligned to the genome on the positive strand; reads map to “-”: number of reads aligned to the negative strand of the genome.

**Figure S1**


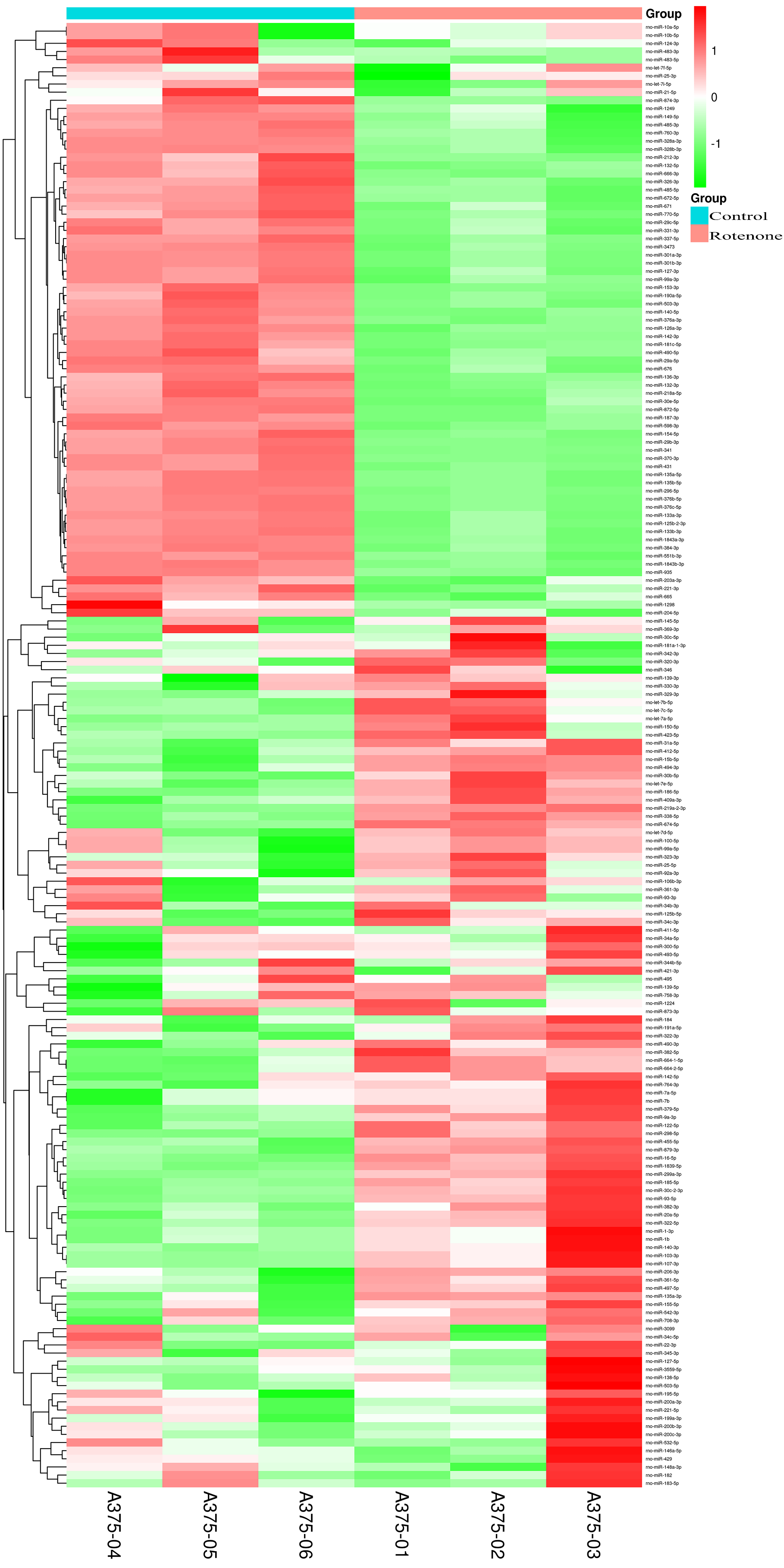


Heat map of differential miRNA expression between control and PD bdsEVs. Next-generation sequencing (NGS) was used to obtain gene expression data on Illumina NovaSeq6000 platform. These values are expressed in reads per kilo, based on a normalized log2-conversion count of per million mapped reads (RPKM). Upregulated and downregulated transcripts are indicated by red and green, respectively.
